# Supplementary material for: Analysis of PPARγ Signaling Activity in Psoriasis
Source: Int J Mol Sci. 2021 Aug 10;22(16):8603. doi: 10.3390/ijms22168603 (PMC8395241; doi:10.3390/ijms22168603)
Supplement: Supplementary file 1 [file ijms-22-08603-s001.zip › Supplemental materials_Analysis of PPARg signaling activity in psoriasis/Pathway models/Models images and html files/PPARG signaling after laser treatment/1399497.html]

laser treatment


# Treatment laser treatment

|  |  |
| --- | --- |
| URN | urn:agi-treatment:laser%20treatment |
| Total Entities | 0 |
| Connectivity | 0 |
| Name | laser treatment |

---

|  |  |
| --- | --- |
| Pathway | PPARG signaling after laser treatment |

---
